# Characteristics of ambulatory anticoagulant adverse drug events: a descriptive study

**DOI:** 10.1186/1477-9560-8-5

**Published:** 2010-02-18

**Authors:** Andrea L Long, Lisa Bendz, Monica M Horvath, Heidi Cozart, Julie Eckstrand, Julie Whitehurst, Jeffrey Ferranti

**Affiliations:** 1 Duke University Health System, Duke Health Technology Solutions, 2424 Erwin Road, Durham, NC, USA; 2 Duke University Hospital, Department of Pharmacy, Erwin Road, Durham, NC, USA

## Abstract

**Background:**

Despite the high frequency with which adverse drug events (ADEs) occur in outpatient settings, detailed information regarding these events remains limited. Anticoagulant drugs are associated with increased safety concerns and are commonly involved in outpatient ADEs. We therefore sought to evaluate ambulatory anticoagulation ADEs and the patient population in which they occurred within the Duke University Health System (Durham, NC, USA).

**Methods:**

A retrospective chart review of ambulatory warfarin-related ADEs was conducted. An automated trigger surveillance system identified eligible events in ambulatory patients admitted with an International Normalized Ratio (INR) >3 and administration of vitamin K. Event and patient characteristics were evaluated, and quality/process improvement strategies for ambulatory anticoagulation management are described.

**Results:**

A total of 169 events in 167 patients were identified from December 1, 2006-June 30, 2008 and included in the study. A median supratherapeutic INR of 6.1 was noted, and roughly half of all events (52.1%) were associated with a bleed. Nearly 74% of events resulted in a need for fresh frozen plasma; 64.8% of bleeds were classified as major. A total of 59.2% of events were at least partially responsible for hospital admission. Median patient age was 68 y (range 36-95 y) with 24.9% initiating therapy within 3 months prior to the event. Of events with a prior documented patient visit (n = 157), 73.2% were seen at a Duke clinic or hospital within the previous month. Almost 80% of these patients had anticoagulation therapy addressed, but only 60.0% had a follow-up plan documented in the electronic note.

**Conclusions:**

Ambulatory warfarin-related ADEs have significant patient and healthcare utilization consequences in the form of bleeding events and associated hospital admissions. Recommendations for improvement in anticoagulation management include use of information technology to assist monitoring and follow-up documentation, avoid drug interactions, and engage patients in their care.

## Background

Because the majority of health care is delivered in the ambulatory care setting, the fact that adverse drug events (ADEs) in outpatients are suspected to occur at a greater frequency than in the inpatient setting [1-3] is a cause for concern. Although the number of studies on ADEs in ambulatory care has grown [3-5], the available information on the nature of these events remains limited. Detection of ADEs varies with the methodology used [2,6]. Also, the vagaries of outpatient settings--where patients oversee the majority of their own daily care and medication use, healthcare visits are periodic, and evidence of errors may go undocumented--further limits ADE detection. Despite the serious nature of these events, which can potentially result in hospitalization, permanent injury, or death, many are in fact preventable [7,8]. While commonly implicated medication classes include cardiovascular drugs, analgesics, hypoglycemic agents, and anti-infectives, several recent studies also implicate anticoagulants as a substantial source of outpatient ADEs and ADEs leading to hospitalizations [3,9-13].

Warfarin is an oral anticoagulant used to prevent and treat thromboembolism, and its use has increased substantially over time, largely because of its indication for atrial fibrillation in an aging population [14,15]. Due to the variable dose-response relationship of warfarin among patients, a narrow therapeutic index, and the potential for numerous drug and dietary interactions, warfarin requires ongoing monitoring during therapy [14]. Monitoring the International Normalized Ratio (INR), a measure of warfarin's effect on clotting factors and the blood's propensity to clot, is essential for maintaining the drug within its narrow therapeutic window. Bleeding events are a significant cause of morbidity and mortality with warfarin therapy [7,15], and the risk of bleeding is largely influenced by the INR value [14]. Safety issues related to anticoagulant use have recently been reexamined by many healthcare systems in light of the Joint Commission's new anticoagulation National Patient Safety Goal 3E requiring action to "reduce the likelihood of patient harm associated with the use of anticoagulation therapy [16]."

Given the seriousness of warfarin-related events in outpatients, we sought to evaluate warfarin-related ADEs and the patient population within the Duke University Health System (DUHS) in which they occurred. Our primary objective was to identify the common characteristics of warfarin-related ADEs in admitted ambulatory patients.

## Methods

### Setting

Duke University Hospital (DUH) is a 1019-bed, tertiary-care teaching hospital with approximately 36,000 patient admissions annually. DUH is part of a greater health system where warfarin therapy is managed by a variety of providers, including primary care clinicians, hematologists, and cardiologists, as well as anticoagulation clinics dispersed amongst these divisions. As part of a comprehensive medication safety program, DUH operates computerized adverse drug event surveillance (ADE-S), an automated trigger tool that scans inpatient clinical data records against clinical rule logic indicative of ADEs or potential ADEs (pADEs) [6,17]. Three clinical pharmacists (κ ≥ 0.88) evaluate each trigger alert by manual chart review to confirm and document adverse event occurrence. Each event is assigned a severity score using the DUH Severity Index [17] and a causality score using the Naranjo algorithm [18].

### Data collection and analysis

Our study group included all adult (age ≥ 18 y) DUH admissions from December 1, 2006-June 30, 2008 for whom outpatient warfarin usage created a bleeding risk (supratherapeutic INR) that was mitigated by inpatient vitamin K administration. These admitted patients were identified using the ADE-S "vitamin K and INR >3" trigger rule. To be included in our analysis, event causality must be ≥ 5 and event severity ≥ 3, meaning patient harm occurred with causality scoring indicating a "probable" or "definite" likelihood of drug involvement. Warfarin administration must have occurred outside of the hospital. DUH admission may have been partly due to coagulopathy. We did not include instances in which a patient presented to the DUH emergency department with a high INR but was not admitted to the hospital. ADEs were defined as any event where bleeding was noted; pADEs were defined as all other events where a supratherapeutic INR was corrected but no bleeding was documented. Instances without history of warfarin administration (e.g., INR elevated due to a disease process) or in which anticoagulation reversal was intentional (e.g., planned procedure pending correction of the INR) were not considered ADEs or pADEs.

Patient demographics, medical history, and hospital visit data were collected from the electronic health record (EHR) and hospital billing system either at the time of event review or retrospectively upon event reexamination. Encounter characteristics included highest INR, last available INR prior to hospitalization, contributing factors, admission reason, bleeding site, concomitant use of medications with bleeding risk, use of additional reversal agents, and hospitalization costs. Major bleeds were classified according to published criteria: fatal bleeding; intracranial, ocular, articular, or retroperitoneal bleeding; surgery or angiographic intervention to stop bleeding; a decrease in hemoglobin levels of ≥ 2 g/dL; and transfusion of ≥ 2 units of blood [19]. Minor bleeding included all other cases of bleeding.

Collected patient characteristics included age, insurance coverage type, indication for anticoagulation, duration of warfarin therapy, date of most recent DUHS healthcare visit, and date of most recent DUHS healthcare visit where anticoagulation therapy was addressed or a follow-up plan was documented. Determination of the provider group responsible for warfarin therapy management was not consistently available in the EHR. Insurance type was collected to capture whether patients without insurance experience a greater volume of adverse events. Collection of previous healthcare encounter information was limited to visits with dictated notes available through the EHR. We considered anticoagulation therapy to be "addressed" if either an INR was measured or a medical note mentioned warfarin management by any provider. We defined "follow-up" as either documented plans to adjust or maintain the warfarin regimen or plans for follow up on INR monitoring.

Descriptive statistics were used to analyze the adverse event and patient characteristic data (JMP 8.0, SAS, Cary, NC, USA). The nonparametric Wilcoxon rank-sum test was used to compare non-normal continuous data. This study was approved by the Duke University Institutional Review Board (Duke University, Durham, NC, USA).

## Results

From December 1, 2006-June 30, 2008, DUH recorded 373,402 admissions, triggering 1444 alerts in the ADE-S system from the "vitamin K and INR >3" rule. Of these, clinical pharmacist reviewers identified 169 outpatient warfarin-related events in 167 patients. A total of 88 (52.1%) had documented evidence of bleeding or patient harm and were considered ADEs; 81 (47.9%) did not document patient harm and were considered pADEs, although these pADEs could have resulted in bleeding events. Two events involved overdoses of warfarin. One of these was an intentional overdose of multiple medications. Despite an INR >10 upon admission, no bleeding was identified; thus, this case was determined to be a pADE. The other overdose case, in a patient with no indication for warfarin, was accidental. The patient mistakenly took several days of a spouse's warfarin because of similarity of packaging with, and proximity to, the patient's medications. Unfortunately, this patient, with an INR of 3.2, suffered a retroperitoneal hemorrhage requiring multiple red blood cell transfusions and hospital admission.

### Event characteristics

Of all ADEs and pADEs, the median highest INR was 6.1 (range 3.1-10.0) (Table 1). The upper limit reportable by the laboratory is an INR of 10. One quarter of events (42/169, 24.9%) involved INRs ≥ 10. A total of 58 (34.3%) of all events had previous INRs electronically available within the previous 2 weeks. Of these, only 39.6% (23/58) were within the generally accepted therapeutic range of 2.0-3.5. Only a third of events (58/169, 34.3%) had explicit documentation of potential contributing factors such as dietary issues (e.g. poor oral intake, diarrhea, vitamin K deficiency), incorrect dosing (e.g. wrong drug dispensed, patient non-compliance), and monitoring (e.g. poor follow-up by either the provider or patient). However, nearly half of these contributing factors (25/58, 43.1%) were due to drug-drug interactions, mainly antibiotics (Table 1). The antibiotics most frequently implicated in 24 events include ciprofloxacin (n = 5), amoxicillin/clavulanate (n = 4), moxifloxacin (n = 3), and trimethoprim/sulfamethoxazole (n = 2).

**Table 1:** Event Characteristics

Characteristic	Total events (n = 169)	ADEs (n = 88)	pADEs (n = 81)
Highest INR, median (range)*	6.1 (3.1-10.0)	5.1 (3.1-10.0)	7.1 (3.3-10.0)
			
Known contributors, No. (%)	58 (34.3%)	29 (33.0%)	29 (35.8%)
Drug-drug interactions	25	12	13
Dietary interaction	30	13	17
Monitoring issue	8	6	2
Dosing issue	6	2	4

*The upper reportable limit of INR laboratory measurement is 10; INRs above this value are reported as >10.

Bleeding occurred in just over half of all identified events (88/169, 52.1%). The most common bleeding sites were gastrointestinal (42/88, 47.7%), intracranial hemorrhage (10/88, 11.4%), hematuria (7/88, 8.0%), and hemoptysis (7/88, 8.0%) (Table 2). Eighty-two of these bleeding incidents were at least partially responsible for the inpatient admission. A substantial proportion of bleeds required red blood cell transfusions (51.1%) and fresh frozen plasma (73.9%) as an additional reversal agent. Major bleeding was evident in more than two- thirds of bleeding events (57/88, 64.8%), with the majority due to gastrointestinal bleeding (33/57, 57.9%), intracranial hemorrhage (10/57, 17.5%) and hematoma (4/57, 7.0%). Four patients with major bleeding required administration of recombinant factor VIIa to assist in coagulation. Concomitant use of medications with risk of bleeding was documented in 51 (51/88, 58%) bleeding events, with aspirin being the most prevalent (46/88, 52.3%) (Table 2).

**Table 2:** Bleeding characteristics (n = 88)

Characteristic	ADEs No. (%)
Use of medications with increased bleeding risk	51 (58.0%)
Aspirin	46
Enoxaparin	6
Clopidogrel	3
Ibuprofen	4
Heparin	1
> 2 of above	9
	
Use of fresh frozen plasma	65 (73.9%)
	
Major bleeds	57 (64.8%)
	
Location of major and minor bleeds*	
Intracranial hemorrhage	10
Gastrointestinal	42
Hematoma	6
Hemoptysis	7
Retroperitoneal	3
Intra-articular	1
Wound/incision site	6
Other	18

*An event may have more than one location of bleeding.

A supratherapeutic INR and/or bleeding incident was either partially or wholly responsible for inpatient admission in 100 (59.2%) outpatient events. These admissions were characterized by a median length of stay (LOS) of 4.8 days (range 0.6-82.7 d), with an associated median hospital cost of $10,419 (range $916-$170,302) per visit. Cost is based on hospital expenses, rather than patient charges or revenue. When subdivided based on whether the patient bled, the median LOS for patients who experienced bleeding was, as expected, significantly longer than for patients without bleeding (LOS 5.3 d [range 0.8-82.7 d] for patients with bleeding; LOS 1.9 d [range 0.7-13.6 d] for patients without bleeding; p < 0.0001). Patients with bleeding also had greater median associated care costs compared with patients without bleeding ($11,526 [range $2,451-$170,302] vs. $2,813 [range $916-$23,582]; p < 0.0001).

### Patient characteristics

The median age of patients involved in ambulatory anticoagulation ADEs and pADEs was 68 y (range 36 y-95 y) (Table 3). All patients were insured, with 78.1% having either private insurance or a combination of Medicare and private insurance (Table 3). Atrial fibrillation (54.4%), deep vein thrombosis (27.2%), valve replacement (13.6%), and pulmonary embolism (15.4%) were the most common indications for anticoagulation. Duration of warfarin anticoagulation could only be determined in 68.6% of events (116/169); more than half (53.4%, 62/116) of these involved patients on warfarin for >12 months, with 36.2% (42/116) newly started on warfarin ≤ 3 months prior to the event (Table 3).

**Table 3:** Patient characteristics

Characteristic	Total events (n = 169)	ADEs (n = 88)	Potential ADEs (n = 81)
Age, mean +/- SD	68.1 +/- 13.2 (range 36-95)	69.5 +/- 13.5 (range 37-90)	66.6 +/- 12.8 (range 36-95)
			
Insurance, No. (%)			
Private	32 (18.9%)	19 (21.6%)	13 (16.0%)
Private/Medicare	100 (59.2%)	52 (59.1%)	48 (59.3%)
Private/Medicaid	3 (1.8%)	0	3 (3.7%)
Medicare	12 (7.1%)	7 (8.0%)	5 (6.2%)
Medicare/Medicaid	14 (8.3%)	7 (8.0%)	7 (8.6%)
Medicaid	8 (4.7%)	3 (3.4%)	5 (6.2%)
			
Indication, No. (%)			
Atrial fibrillation	92 (54.4%)	51	41
Deep vein thrombosis	46 (27.2%)	19	27
Pulmonary embolism	26 (15.4%)	13	13
Stroke	23 (13.6%)	15	8
Valve replacement	23 (13.6%)	11	12
Hypercoagulable	11 (6.5%)	3	8
Other	10 (5.9%)	4	6
Indeterminate	1 (0.6%)	0	1
None	1 (0.6%)	1	0
			
Duration of warfarin therapy, No. (%)			
<1 mo	25 (14.8)	12 (13.6)	13 (16.0)
1-3 mos	17 (10.1)	8 (9.1)	9 (11.1)
4-6 mos	9 (5.3)	3 (3.4)	6 (7.4)
7-12 mos	11 (6.5)	8 (9.1)	3 (3.7)
1-5 y	29 (17.2)	13 (14.8)	16 (19.8)
> 5 y	25 (14.8)	15 (17.0)	10 (12.3)
Indeterminate	52 (30.8)	28 (31.8)	24 (29.6)
No prescribed warfarin	1 (0.6)	1 (1.1)	0
			
Seen at Duke clinic or hospital within 30 d, No. (%)*	115 (73.2%)	52 (65.0%)	63 (81.8%)
			
Anticoag. therapy addressed within 30 d, No. (%)*	90 (57.3%)	43 (53.8%)	47 (61.0%)

*Denominator limited to events with a prior encounter note present in the EHR:

Total events = 157, ADEs = 80, pADE = 77.

Nearly all events (157/169, 92.9%) involved patients with at least 1 prior healthcare visit, as evidenced by an inpatient or outpatient provider note in the EHR. The median time since previous encounter was 15.8 days (range 0.6-2994.7 d), with the majority of previous encounters being ambulatory visits (114/157, 72.6%). Nearly three-fourths of events (115/157, 73.2%) involved patients who were last seen by a DUHS provider in the previous month; 26.1% (30/115) were recently hospitalized. All hospitalized patients had their anticoagulation therapy addressed, as evidenced by INR monitoring and/or documentation of management in the discharge summary. However, only 76.7% of these hospitalizations had anticoagulation follow-up plans documented in the discharge summary. Among patients whose last encounter occurred in the ambulatory care setting in the previous month (85/115, 73.9%), only 56.5% had documented evidence that anticoagulation therapy was addressed, and only 45.9% had documented follow-up plans.

Since we were unable to confirm whether the last seen provider was responsible for monitoring warfarin therapy, we also evaluated whether these assessments took place at any visit in the past month. Of 115 events in which a patient had a documented visit note in the EHR within the past month, 90 (78.3%) addressed anticoagulation therapy and 69 (60.0%) had follow-up plans noted in at least one healthcare visit note. Not all medical services share primary responsibility for anticoagulation therapy management. However, among the services noted as failing to address anticoagulation therapy in the previous month, cardiology, hematology, and medicine, which often oversee warfarin therapy management based on corresponding indications, accounted for a total of 32.0% (8/25).

## Discussion

In this study, we used computerized ADE surveillance of hospitalized patients, coupled with targeted chart review, to identify 169 warfarin-related ADEs and pADEs that occurred in the DUHS ambulatory environment. Our descriptive analysis demonstrates that anticoagulant ambulatory events have a significant clinical impact on patients and contribute significantly to healthcare costs.

Although the rate of ADEs can vary depending on data collection methodology, several studies have reported that anticoagulants, including warfarin, are among the medication classes most frequently associated with ambulatory ADEs and ADEs requiring hospital admission [3,9-13]. Using a computer-based event detection application, Jha and colleagues identified hospital admissions due to ADEs. Anticoagulants were implicated in 9% (7/76) of these ADEs; however, warfarin was the most common individual agent identified in all events [11]. In a review of elderly patients by Gurwitz et al, anticoagulants accounted for 8% of ambulatory ADEs, the fifth most common medication class involved in events [10]. As part of the National Electronic Injury Surveillance System-All Injury Program, trained coders from nine participating hospitals identified ADEs noted in emergency department records. Anticoagulants were associated with 4.7% of ED visits; more strikingly, they were the third leading cause (15.4%) of ADEs resulting in hospitalization. Together, warfarin and insulin accounted for 16% of all outpatient ADEs and 33% of outpatient ADEs in patients aged ≥ 50 years [9].

Not only is warfarin involved in a large proportion of ambulatory ADEs, but its effect on patient outcomes is also substantial. In our study, the median highest INR at the time of the event was 6.1, and more than half (52.1%) of these cases resulted in a bleeding event. The majority (64.8%) were defined as major bleeds, with intracranial hemorrhages accounting for 17.5% of these. The intensity of warfarin therapy is known to be strongly associated with bleeding risk, and an INR ≥ 4.5 is the single greatest risk factor for bleeding [14].

Patients and health systems are also affected by hospital admissions stemming from these warfarin-related events. Due to the nature of our surveillance system, all events were identified in patients already admitted to the hospital for reasons that could include coagulopathy, other diagnoses, or both. Interestingly, more than half (59.2%) of admissions were due at least in part to correction of coagulopathy. Budnitz et al reported on ambulatory ADEs identified via surveillance of ED records in geriatric patients. Among warfarin-related ADEs, not only did 73% result in bleeds, but 44.2% of cases required hospitalization [13]. Other studies have noted the propensity for warfarin-related ADEs to lead to hospitalization [9,11].

Such potentially avoidable hospitalizations can be very costly to the healthcare system. In our study, for each admission due to coagulopathy regardless of bleeding, the median cost of care was $10,419, with a median LOS of 4.8 d. Of course, other diagnoses and complicating factors may have influenced both cost of care and LOS, but a broad generalization suggests that if the ADEs and pADEs had not occurred, more than half of the hospital admissions could have been avoided, saving the hospital and payers nearly $1.7 million over 19 months. Moreover, 73.9% of bleeding events required fresh frozen plasma to rapidly reverse the INR, and 51.1% required blood transfusions. Similarly, Jha and colleagues noted 76 ambulatory ADEs led to hospital admissions totalling 738 patient days, with a median 5-day LOS and an average cost of $16,177 per visit [11]. Interestingly, 24 patients in our study experienced a total of 28 additional warfarin-related ADEs identified by ADE-S either before or subsequent to the study period. Ten of these events occurred within 6 months following the study. These potentially avoidable warfarin-related events result in significant healthcare costs to the DUHS, and such problems are likely to be found in other organizations as well.

Quality improvements in ambulatory care can be accomplished by strengthening the information technology (IT) infrastructure [20]. The main process areas that should be targeted include prescribing and monitoring, since errors in these processes respectively account for 41.0%-64.7% and 26.0%-72.7% of preventable ambulatory ADEs [3,10,21]. For warfarin-related events in this study where contributing factors could be identified retrospectively, we found drug interactions, particularly between antibiotics and warfarin, to be most common. Antibiotics are known to contribute to deviations in INR; thus, initiation of such medications in outpatients without close monitoring may result in supratherapeutic INRs and possible harmful outcomes. Ambulatory e-prescribing with drug interaction checking can help reduce medication errors [22,23]. In addition, ambulatory EHRs can have further additive benefits by providing electronically-available provider notes and medication reconciliation. In this study, 73.2% of the events for which a prior record existed in our EHR occurred in patients seen at an inpatient or outpatient DUHS healthcare visit within the previous 4 weeks; however, 40.0% did not have a documented follow-up plan within that same period. EHRs can help bridge the communication gap during transitions in care and assist providers with decision-making and lab test follow-up [20]. This is extremely important not only for warfarin use, where monitoring is critical, but also applies broadly to clinical care given the segmented nature of health care.

However, the impact of quality improvement strategies for the safe management of warfarin therapy may be most felt in terms of fostering patient empowerment and engaging patients in the ownership of their care. Patient nonadherence has been reported as being among the most common drug therapy problems associated with hospital admissions [3]. In our study, evidence of non-compliance was limited to specific documentation by providers and thus the true rate of nonadherence is likely under-reported. Furthermore, the infrastructure of the ambulatory care environment is typically segmented and logistically complex, with patients often forced to seek their care from various providers or specialists, pharmacies, and laboratories spanning multiple health systems.

For these reasons, it is clearly essential that patients be equipped with the knowledge and understanding of their role in their own care. Recognizing this, the Joint Commission instituted National Patient Safety Goal 13, which encourages "...patients' active involvement in their own care as a patient safety strategy" [16]. Patient education is also encouraged in the CHEST guidelines, and at least one study reported lower warfarin-related hospitalizations for bleeding in patients who received education regarding warfarin therapy [14,24].

Health portals or personal health records can also be leveraged to encourage active patient involvement in improving drug safety. Health portals not only provide education via access to consumer medication and health knowledge resources, but also allow access to patients' medical information (e.g., displaying current medication lists and laboratory values) [25]. Moreover, interactive portals could permit patients to enter information, such as INR values or medication changes, obtained from various facilities, to maintain an accurate, up-to-date health record [25,26]. The full potential of portals has yet to be explored, but thus far, patients report satisfaction with using many of their features [26-28].

Though not suitable for all patients, home monitoring of INR using point-of-care testing (POCT) also remains a viable option for patient involvement [29-32]. Although 73.2% of our events were reported in patients who had a healthcare visit within the previous month, INR elevations still occurred. Deviations in warfarin control may be due to events occurring between healthcare visits that the patient may be first to recognize and mitigate if given proper knowledge and tools. Furthermore, use of POCT has shown reductions in bleeding and thromboembolic complications [33].

Additional improvements in warfarin dosing may also be made possible by the clinical application of pharmacogenomic guided therapy [34-36]. Single nucleotide polymorphisms (SNPs) in the *CYP2C9 *and *VKORC1 *genes have been associated with a wide variability in patient response to warfarin dosing and a potentially greater risk for adverse events [34,36]. A randomized controlled trial of the clinical safety and efficacy of genotype-guided warfarin dosing (the European Genetics of Anticoagulant Therapy Trial [EU-PACT]) is currently under way [37].

Our study has several limitations that should be acknowledged. We only detected events in patients admitted to the hospital and did not capture warfarin-related events requiring visits to the ED only, urgent care, or other outpatient facilities. In addition, we did not capture warfarin-related events for which the INR value was within the therapeutic range due to the conditions of the surveillance rule logic. Interestingly, Oake et al noted that half of all bleeding complications in outpatients occurred despite an INR value within therapeutic range [38]. Given our retrospective review, we could only confirm documentation of follow-up plans, but it remains unknown whether the plan was actually discussed explicitly with the patient, or if a plan was verbally communicated and not documented.

## Conclusions

Ambulatory warfarin-related ADEs have significant effects on both patient outcomes and healthcare costs, as evidenced by bleeding outcomes, hospitalizations, and transfusion of blood products. The majority of patients we examined had recently received health care within the DUHS, yet documentation of warfarin follow-up plans was lacking, and warfarin-antibiotic drug interactions were noted. The use of IT to assist in monitoring and follow-up documentation (ambulatory EHRs), for avoidance of drug interactions (electronic prescribing), and to engage patients in their care (personal health portals) should be explored in order to provide safer care for patients receiving warfarin therapy.

## Competing interests

The authors declare that they have no competing interests.

## Authors' contributions

AL and LB contributed to study design, data collection and analysis, as well as drafting of the manuscript. MH assisted with data analysis and revision of the manuscript. JE and JW participated in data collection and event analysis. HC and JF contributed to the conceptual design of the study. All authors read and approved the final manuscript.
